# A habenula-enriched GPCR, GPR151, regulates behavioral sensitivity to inflammation

**DOI:** 10.64898/2026.08.02.742327

**Published:** 2026-08-07

**Authors:** Lupita Rios, You-Hsin Lin, Laura Yuan, Yash Sharma, Hailey Arias, Fizza Jeddy, Sravya Thotakura, Aiden Geleta, Raghav Rajesh, Steven Shabel

## Abstract

**Background:**

Inflammation-associated depression is a subtype of major depressive disorder that is often resistant to conventional pharmacotherapies, which act in a regionally non-specific manner and therefore also produce unwanted side effects. Here we test GPR151, an orphan GPCR associated with inflammation and highly expressed in the habenula—a region linked to negative valence and depression—as a therapeutic target for inflammation-associated depression.

**Methods:**

We integrated mouse and human habenular expression analyses with genetic loss-of-function and adult habenular re-expression approaches in mice. Gpr151 knockout mice and littermate controls were exposed to lipopolysaccharide (LPS) inflammatory challenge and assessed for stress coping and motivated behavior, body weight loss, and peripheral immune activation. To test whether adult habenular GPR151 expression is sufficient to restore inflammation-associated behavioral vulnerability, GPR151 was re-expressed in the habenula of knockout mice.

**Results:**

GPR151 was exceptionally enriched in the habenula and showed conserved topographic organization and similar expression relationships with habenular marker genes in mice and humans. Following LPS challenge, male Gpr151 knockout mice showed reduced passive coping despite body weight loss and immune activation comparable to littermate controls. Adult habenular GPR151 re-expression increased LPS-induced amotivation in male knockout mice without increasing LPS-induced weight loss or immune activation. Female Gpr151 knockout mice also showed reduced passive coping after LPS challenge; however, habenular GPR151 re-expression was insufficient to increase LPS-induced amotivation in females.

**Conclusions:**

These findings identify GPR151 as a conserved, regionally enriched regulator of behavioral sensitivity to inflammatory challenge and support GPR151 as a candidate therapeutic target for inflammation-associated depression.

## Full Text Availability

The license terms selected by the author(s) for this preprint version do not permit archiving in PMC. The full text is available from the preprint server.

